# Production and Shelf-Life Study of Probiotic Caja (*Spondias mombin* L.) Pulp Using *Bifidobacterium animalis* ssp. *Lactis* B94

**DOI:** 10.3390/foods11131838

**Published:** 2022-06-22

**Authors:** Thais Jaciane Araujo Rodrigues, Aline Pacheco Albuquerque, Antônio Vinícius Silva de Azevedo, Layanne Rodrigues da Silva, Matheus Augusto de Bittencourt Pasquali, Gilmar Trindade de Araújo, Shênia Santos Monteiro, Wanessa Dayane Leite Lima, Ana Paula Trindade Rocha

**Affiliations:** 1 Academic Unit of Agricultural Engineering, Federal University of Campina Grande, Campina Grande 58428-830, Brazil; thaisjaraujo@hotmail.com (T.J.A.R.); aline-quimicaindustrial@hotmail.com (A.P.A.); ana_trindade@yahoo.com.br (A.P.T.R.); 2 Academic Unit of Food Engineering, Federal University of Campina Grande, Campina Grande 58428-830, Brazil; azevedovinicius_ufcg@hotmail.com (A.V.S.d.A.); laayanne1@gmail.com (L.R.d.S.); gilmartaraujo@outlook.com (G.T.d.A.); wanessadayane@gmail.com (W.D.L.L.); 3 Center for Technology and Natural Resources, Federal University of Campina Grande, Campina Grande 58428-830, Brazil; shenia-monteiro@hotmail.com

**Keywords:** functional food, fermentation, growth kinetics, stability study

## Abstract

The highly nutritional caja fruit (*Spondias mombin* L.) is an accessible source of vitamins and antioxidants that are indispensable for the human diet. The objective of the present work was to study the production of a probiotic caja pulp using *Bifidobacterium animalis* ssp. *lactis* B94. Firstly, a kinetic study was performed on the fermentation of the caja pulp with *Bifidobacterium animalis* ssp. *lactis* B94 to determine the optimum conditions of the process. Growth kinetics revealed that the ideal time for ending the fermentation would be at 22 h because it corresponds to the end of the exponential phase. Both the whole pulp and the probiotic pulp were characterized for pH, acidity, total soluble solids, water content, phenolic content, reducing carbohydrates, ascorbic acid, and total carotenoids. Physicochemical characterization revealed similar results between the whole and the probiotic pulp. The stability test demonstrated that the probiotic pulp is stable and preserved the probiotic attributes of the final product. In conclusion, our results reveal that caja pulp can be considered a favorable medium for the *Bifidobacterium animalis* ssp. *lactis* B94 growth and consequently can be explored biotechnologically for new food products.

## 1. Introduction

Fruits are an integral component of the human diet, a source of micronutrients such as vitamins, minerals, and phytochemicals that offer nutritional and health benefits [1]. In Brazil, the biodiversity of native fruits represents an important source of vitamins. The cultivation and consumption of especially exotic fruits are increasing in local and international markets, holding a potential interest due to the recognition of their nutritional and therapeutic value [2]. In addition, the appreciation of native exotic fruits brings benefits to family farming and consequently contributes to the improvement of global food and nutritional security [3] due to the high content of bioactive molecules present in the fruits. These bioactive molecules, when integrated into the human diet, can act on the biological defenses, enhance physical activities conditions, prevent, retard, or even treat illnesses

The caja (*Spondias mombin* L.) is a native fruit of tropical areas of America, Asia, and Africa, belonging to the Anacardiaceae family [4]. While fruit is usually consumed fresh, the scientific community and the food industry have been seeking to develop several derived products, such as ice cream and juices fermented by probiotics, for better use of production [5]. Nutritionally, caja has numerous metabolites with functional properties and is a natural source of carotenoids, vitamin A and vitamin C, minerals such as potassium and copper, in addition to containing a significant amount of bioactive compounds and dietary fibers [4]. The antioxidant properties present in the pulp of the caja were related to several beneficial health effects, such as cardiac remodeling after myocardial infarction [6], promoted healing of the chronic ulcer, regeneration of the gastric mucosa, and restoration of mucus levels in glandular cells [7]. The caja fruit also has a perceptive aroma, sweet-sour taste, and intense coloration. Due to its favorable sensorial attributes, caja has high acceptability among consumers [8]. These characteristics have stimulated the food industry to focus on studies for increasing the nutritional and functional value of caja products without losing their fundamental properties. Despite being a source of nutrients and compounds beneficial to health, during the harvest, a large percentage of this fruit is wasted due to its high water content, high respiration rate, and rapid ripening process, which is a major challenge for the production of fruit, since postharvest losses are high [9].

Several fruit conservation technologies have been studied over the years to reduce postharvest losses of tropical fruits. One of the solutions to this challenge is the application of fermentation technology. Fermentation is a biotransformation process in which microorganisms use energy derived from the catabolization of carbohydrate-rich substrates for their growth [10]. Recently there has been a renewed interest in fermentation, especially using lactic acid bacteria, due to the probiotic characteristics that can confer some health benefits, including antiallergic, antihypertensive, anti-inflammatory, prevention, and control of chronic diseases such as diseases: cardiovascular disease, type II diabetes, obesity, and cancer [11]. Although dairy is traditionally considered the best food matrix for fermentation by lactic acid bacteria, notably, fruit juices have been reported as a novel and suitable medium for microorganisms [12,13].

Development of probiotic foods through fermentation requires a complex approach, principally because different factors can affect the probiotic cell viability in foods, such as the probiotic stirp, pH, dissolved oxygen content, metabolites such as lactic or acetic acid, buffer capacity, storage temperature, additives, and the own food medium [14]. In addition, during the preparation and storage of the probiotic product, it is necessary to maintain the viability of the microorganisms high so that the health benefits of the host can be obtained [12,14]. It has been reported that approximately 100 g/day of probiotic products must be consumed regularly to deliver about 9 viable Log CFU in the gut [15]. In this scenario, the choice of food matrix and starter culture is essential to favor the aggregation of probiotics in foods.

The initial strains of the *Lactobacillus* genus are recognized as the most adapted to substrates of plant origin [12]. Although studies with *Bifidobacterium* are mostly limited to the use of species in dairy products, the study of the adaptation of *Bifidobacterium* species in fruit-based food matrices should be investigated as a potential probiotic used in the development of functional foods and appreciation of exotic fruits. Therefore, studies that address the effects of probiotic strains on the profile of phenolic compounds and antioxidant activity can provide relevant information for the development of fruit derivatives with high-value content [16].

Given the accelerated innovations in probiotic foods, here we investigate the development of a probiotic food through the fermentation of caja pulp by *Bifidobacterium animalis* spp. *lactis*, carrying out a study to evaluate cell growth, retention of compounds with biological activity beneficial to health, and probiotic strain survival and physicochemical characteristics of fermented caja pulp during cold storage.

## 2. Materials and Methods

### 2.1. Preparation of Caja Pulp

Caja fruit (*Spondias mombin* L.) was purchased at the local market of Campina Grande (Paraiba—Brazil), handpicked at the ripe maturation stage, sanitized with sodium hypochlorite solution, and rinsed with running water. Caja pulp was produced using a semi-industrial pulper (model 670, BONINA, Itabuna Brazil). After pulp extraction, the samples were then stored at −18 °C before use.

### 2.2. Growth Kinetic and Caja Pulp Fermentation

The pH of the caja pulp was firstly adjusted to 7.0 using an aqueous solution of sodium hydroxide (NaOH). Sodium hydroxide was used because it is considered a food acidity regulator [17]. A control medium composed of Man Rogosa Sharpe—MRS (Kasvi©, São José do Pinhais, PR, Brazil) culture and 0.05% L-cysteine (SYNTH©, Diadema, SP, Brazil) was produced. The pulp and the control medium were pasteurized for 20 min using a heated bath at 65 °C. After cooling, the pulp and the control medium were inoculated with *Bifidobacterium animalis* ssp. *lactis* B94 (DELVO^®^PRO LAFTI B94, DSM Food Specialties, Moorebank, Australia) lyophilized directly into pulp and control medium. Based on preliminary tests (unpublished data), it was observed that the activation of the bacteria directly in the fermentation medium resulted in greater growth in a shorter process time; therefore, the methodology of direct inoculation of the bacteria in the fermentation medium was adopted. The initial number of bacteria in the inoculum was 10 Log CFU/mL. A high initial number of the inoculum was considered in the study to obtain high probiotic viability after the storage period. This procedure was performed in a laminar flow cabinet. Both fermentations were kept at (35 ± 2) °C and monitored for 30 h. Every three hours, the number of viable cells was determined, along with pH and acidity, following the Association of Official Analytical Chemists (AOAC) methodology [18]. The growth kinetic follow-up was performed in the caja pulp and the control medium.

### 2.3. Determination of the Number of Viable Cells

The probiotic bacteria count was performed after serial dilution of the fermentation broth samples with peptonized water at a concentration of 0.1%, according to the methodology from the International Dairy Federation [19]. Petri dishes with a growth medium composed of Agar MRS added with L-cysteine at 0.05% g/L [20] were inoculated and incubated at 37 ± 2 °C for 72 h in an anaerobiosis jar with an oxygen removal system. The results were expressed in the colony-forming unit (Log CFU/mL).

### 2.4. Kinetic Parameters

Kinetic parameters of the growth of *Bifidobacterium animalis* ssp*. lactis* in caja pulp in the control medium (MRS broth) were calculated by the Equations (1) and (2):

(1) $$\ln\left(\frac{X_{\max}}{X_{i}}\right){=\mu }_{\max}\left({t\;-\;t}_{i}\right)$$

(2) $$\mu_{\max}=\frac{\ln2}{t_{g}}$$

where X_i_ is the number of bacterial cells at the beginning of the exponential growth phase (Log CFU/mL), t_i_ is the time corresponding to the beginning of the exponential growth phase (h), and t_g_ is the generation time (h).

### 2.5. Physicochemical Characterization of the Whole and Probiotic Caja Pulp

The analytical procedures for the physicochemical characterization were performed according to the methods proposed by the Association of Official Analytical Chemists [18], such as pH, with reading performed in a pH meter previously calibrated with buffer solutions 7.0 and 4.0 at a temperature of 20 °C. The acidity by titration of the sample with 0.1 N sodium hydroxide solution, using phenolphthalein as an indicator. The total soluble solids, using a portable refractometer. The water content and total solids are in an oven at 105 °C until the weight is constant. The analysis of reducing carbohydrates was based on the method proposed by Miller [21], using Reactive DNS, and the absorbance readings were performed with the sample at room temperature in a spectrophotometer at 575 nm. The content of ascorbic acid was determined by the method using the reagent 2,6-dichlorophenol-indophenol, as described by Keller and Schwager [22]. The total phenolic content was determined according to the method described by Singleton and Rossi [23], using the Folin-Ciocalteau reagent. The total carotenoid content was determined according to the Lichtenthaler [24], using 80% acetone to obtain the extracts. All analyses were performed in triplicate.

### 2.6. Total Reactive Antioxidant Potential (TRAP) and Total Antioxidant Reactivity (TAR) of the Whole and Probiotic Pulp

The 2,2-Azobis (2-amidinopropane) dihydrochloride (AAPH) solution, with a final concentration of 120 mM, was prepared by adding the AAPH reagent in glycine buffer 100 mM, pH 8.6 (20 mL final volume), followed by the addition of luminol (4 μL, final concentration of 0.001 mM) in dark conditions. The solution system was then stabilized for 2 h before the first reading. Different concentrations of probiotic pulps were added, and the luminescence produced by the free radical reaction was quantified in a liquid scintillation detector (Wallac 1409, Perkin-Elmer, Boston, MA, USA) for 2 h. The system quantified the chemiluminescence emitted by AAPH thermolysis alone. The data were transformed into an area under the curve (AUC) using the GraphPad Prism (San Diego, CA, USA; version 8.0, www.graphpad.com (accessed on 1 August 2021)) [25]. Total antioxidant reactivity (TAR) was determined with the same experiment [26].

### 2.7. Probiotic Pulp Stability during Storage

The probiotic pulp was stored under refrigeration (4 ± 2 °C) for 28 days. The storage period was defined based on previous literature [27,28]. On days 1, 7, 14, 21, and 28, samples were taken to assess the pH, acidity, and total soluble solids (°Brix). Additionally, the number of viable cells was also determined for each time point. The evaluation took place as previously described in Item 2.3, for the medium based on caja pulp and for the control medium (MRS broth), with the addition of 0.05% of L-cysteine.

### 2.8. Statistical Analysis

Results were assessed using one-way ANOVA at 5% probability, and the significant qualitative responses were submitted to the Student *t*-test using the Minitab Software version 17.0 (www.minitab.com (accessed on 1 August 2021)) [29]. The graphs were made using GraphPad Prism, version 8.0 [25].

## 3. Results and Discussion

### 3.1. Growth Kinetics of in Caja Pulp

**Figure 1:**
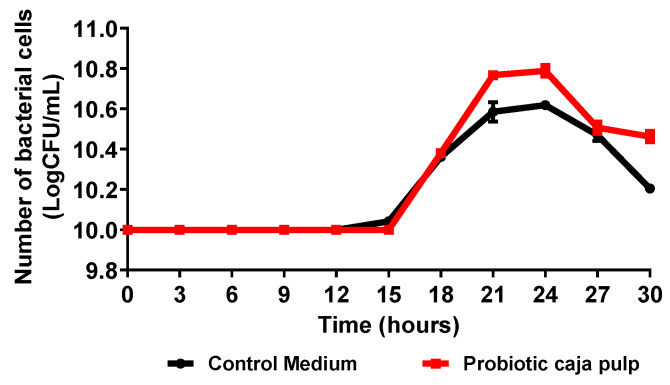
Time course of the number of viable cells during the *Bifidobacterium animalis* ssp. *lactis* B94 fermentation in caja pulp and the control medium (MRS broth).

The time course of *Bifidobacterium animalis* ssp. *lactis* fermentation in caja pulp and the control medium (MRS broth) is presented in Figure 1. Results are expressed as the number of viable cells as a function of time. The time course presents a typical curve for bacterial growth according to the stages proposed by Maier e Pepper [30]. Thus, before the 15 h, a lag stage is observed due to the adaptation of the bacteria to the medium. The lag period observed may be related to the temperature of the medium, pH, and available nutrients. Then, between 15 h and 21 h, the exponential phase is observed, where the maximum growth rate is reached. A stationary phase is observed between 21 h and 24 h, where the bacteria reaches its maximum concentration. At the decline stage, the growth is inhibited due to substrate deficiency, and the bacteria experience oxidative stress. All stages are influenced by medium properties such as the pH, substrate concentration, available nutrients, temperature, and time [31].

Biological parameters for the fermentation process of caja pulp and control medium by *Bifidobacterium animalis* ssp. *lactis* are summarized in Table 1. Initially, the concentration of *Bifidobacterium animalis* ssp. *lactis* was approximately 10 Log CFU/mL; after 24 h of fermentation, an increase was observed to more than 10.79 Log CFU/mL in the caja pulp and 10.62 Log CFU/mL in the control medium. It is noted that caja pulp favored the growth of *Bifidobacterium animalis* ssp. *lactis*, superior to the control medium. The caja pulp showed a maximum growth rate equal to the control medium. However, the generation time was slightly shorter in the fermentation with caja pulp. However, a long period of adaptation of the probiotic culture was observed in both fermentation media. Process conditions such as temperature, pH, and agitation can influence the adaptation time [32]. The caja pulp is nutritionally rich in carbohydrates, vitamins, phenolic compounds, and other components; thus, it is assumed that these nutrients were used by the probiotic culture as a source of energy [33]. Plant matrices are presented in several studies as a promising medium for the growth of probiotic cultures [5,33]. However, growth was affected by the fermentation medium. Solval et al. [34] studied the kinetics of lactic acid bacteria growth in different fermentation media, making a comparison with the MRS medium, and saw that the supplemented medium had a better performance compared to the others, attributing the result to the greater amount of low molecular weight peptides and free amino acids.

**Figure 2:**
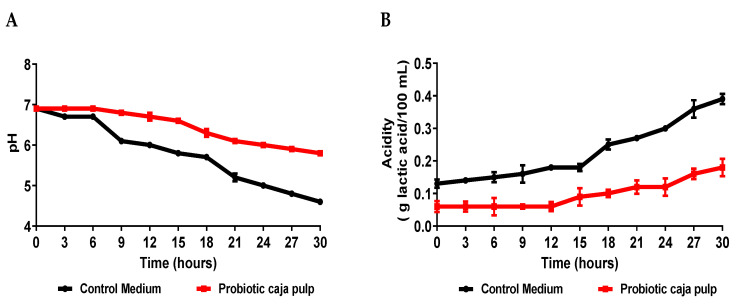
Time course of *Bifidobacterium animalis* ssp. *lactis* B94 fermentation in caja pulp and the control medium. (**A**) pH; (**B**) Acidity.

Fermentation success was related to acid formation. Markkinen et al. [35] showed that the initial increase in lingonberry pH before fermentation is likely necessary, despite the loss of most of its antimicrobial properties due to deprotonation. The pH decreased during the fermentation (Figure 2A). The main metabolic pathway of the lactic bacteria produces lactic acid. This acid molecule works as a preservative during the process and, consequently, on the final product. Therefore, even though lactose is not present on the caja pulp, the lactic acid produced by the bacteria still lowers the pH. This behavior is typical of *Bifidobacterium animalis* ssp. *lactis*, independently of the medium where it grows [15]. Moreover, we believe that *Bifidobacterium animalis* ssp. *lactis* should be similar behavior in human physiology, thus contributing to the reduction in gut pH and stimulating its growth and proliferation in the microbiota [36,37].

While comparing the time course for the number of viable cells and the pH, it is observed that after 24 h of fermentation, the pH becomes less than 5.0. A pH value above 5.0 is critical for the bacteria’s growth. We suggest that at 24 h, the bacteria start its death stage because the medium does not favor its survival. Our results reveal that at 24 h also is possible observing that it reached the maximum number of cells in the probiotic pulp. Similar results observed in the pH can be verified in the acidity (Figure 2B).

Comparing the results presented in Figure 2 for the caja pulp and the control medium, it is noted that the production of acids was higher in the control medium, presenting a curve with a more accentuated decline. Zeybek et al. [38] show that the pH values of the broth cultures of five species of *Bifidobacterium* decreased from their initial value of 7.0 to a minimum of 4.87 in the medium using XOS and the organism *Bifidobacterium animalis* ssp. *Lactis.* Usta-Gorgun and Yilmaz-Ersan [39] reported faster growth and lower pH values of media composed of 2% salep, considered a prebiotic. Therefore, it is assumed that in the control medium, the probiotic found a simple carbon source that was metabolized faster than in the caja pulp. Still, the pulp of caja showed greater growth of *Bifidobacterium animalis* ssp. *lactis*, which indicates that the nutrients present in the fermentation medium were used for cell development. When fermentation substrates are consumed by animals and humans, acidity in the colon decreases depending on the structure and concentration of substrates such as prebiotics [39]. Acidification during the fermentation process results in a reduction in the growth of pathogenic bacteria and an increase in beneficial macrobiotics, composed, for example, of cultures of the *Bifidobacterium* and *Lactobacillus* genera [40].

### 3.2. Physicochemical Characterization of the Whole and the Probiotic Caja Pulp

Table 2 presents the physicochemical characterization of the whole and probiotic caja pulp. The probiotic pulp has a substantially different pH and acidity when compared with the whole pulp due to the pH correction performed before the fermentation. A pH close to neutrality was necessary to guarantee favorable conditions for the bacteria growth within the caja pulp. The pH decreases during the fermentation because the *Bifidobacterium animalis* ssp. *lactis* produces metabolites that decrease the pH as the substrate is consumed [36]. Since acidity is a reverse parameter of the pH, a similar pattern is observed, tough for a lower value when compared to the whole pulp.

The total soluble solids (°Brix), and the total solids (%) of the probiotic pulp, presented lower values when compared to the whole pulp. This result is justified by the fact that the probiotic culture requires substrates for its metabolism during fermentation. Water content does not present a significant change between pulps.

Ascorbic acid content significantly decreased after fermentation. Wang et al. [41] showed that the ascorbic acid content increased in kiwi juice cultivar Xuxiang after fermentation and decreased in kiwi juice cultivar Hongyang after fermentation, both fermented with *Lactobacillus acidophilus* 85 strain. Ascorbic acid is an indicator of fruit quality, and it has been suggested that its stability depends on the redox state, the presence of metal ions, and the pH of the evaluated medium [41,42]. Furthermore, ascorbic acid degradation can be attributed to the presence of ascorbic acid oxidase in citric juice, which may not be fully inactivated by pasteurization treatment [43].

Phenolic compounds are relevant bioactive molecules for the human diet due to their antimicrobial and antifungal effects on the human body. The whole caja pulp is considered rich in phenolic compounds since it presents a concentration of 185 mg/g. When evaluating the data in Table 2, it is noted that the caja pulp after fermentation showed a non-significant tendency toward an increase in the content of phenolic compounds and total carotenoids, but studies such as the one carried out by Wu et al. [44] shows changes in phenolic acid content during the 48 h fermentation of blueberry and blackberry juices fermented by *Lactobacillus plantarum*, *Streptococcus thermophilus* and *Bifidobacterium bifidum* that can be attributed to the probiotic’s potential to metabolize phenolic acids during fermentation. Similar to phenolic compounds, the carotenoids are natural pigments responsible for the yellow, orange, and red coloration of fruits and vegetables. These pigments are not produced by the human body but are present in high concentrations in fruits from the species of the genus *Spondias* due to their characteristic coloring. Despite the non-significant results (*p* > 0.05) for the carotenoid content, we observed an increasing trend after fermentation. Similar results were discussed by Fuente et al. [45] in the study of samples of orange juice fermented by *Lactobacillus brevis* and *Lactobacillus plantarum*.

Another relevant nutritional component is the content of reducing sugars. The reducing sugar content showed a non-significant reduction after fermentation. Studies indicate that fructose, glucose, and sucrose may constitute up to 65% of all soluble matter in fruits of the *Spondia* family [46]. Peng et al. [47] reported in their study that fructose and sucrose were the main sugars used by lactic acid bacteria during fermentation, with a significant reduction in fructose in apple juice fermented by a mixture of *Lactobacillus acidophilus, Lactobacillus plantarum*, and *Lactobacillus fermentum*. Therefore, these findings indicate that during the fermentation of caja pulp, components other than glucose may have been used by *Bifidobacterium animalis* ssp. *lactis*.

### 3.3. Total Reactive Antioxidant Potential (TRAP) and Total Antioxidant Reactivity (TAR) of the Whole and Probiotic Pulp

**Figure 3:**
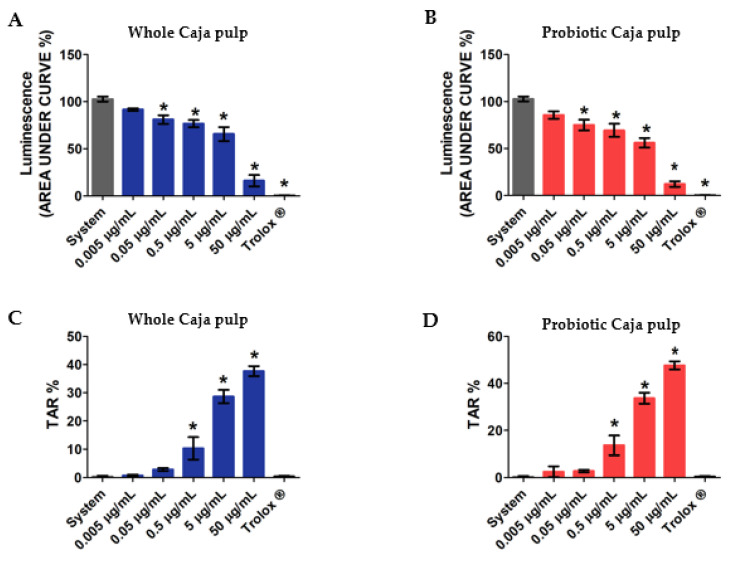
Analysis of total reactive antioxidant potential (TRAP) and total antioxidant reactivity (TAR) of the whole and probiotic caja pulps: (**A**) TRAP whole caja pulp; (**B**) TRAP probiotic caja pulp; (**C**) TAR whole caja pulp; (**D**) TAR probiotic caja pulp. The mean of three replicates (*n* = 3) and the values are expressed as the mean ± SEM. * *p* < 0.05 value describes a comparison through ANOVA. Tukey test was performed.

In Figure 3, our results reveal the total reactive antioxidant potential and the total antioxidant reactivity of both the whole and the probiotic pulp. Each assay used increasing concentrations of whole pulp and probiotic pulp, from 0.005 μg/mL, 0.05 μg/mL, 0.5 μg/mL, 5 μg/mL, to 50 μg/mL. Our assay also evaluated a control group represented by the system and a positive control group with Trolox (analog of hydrophilic vitamin E), which was used as a reference antioxidant. According to different authors. TRAP is commonly used to determine the antioxidant activity of a given compound or groups of compounds in distinct products [48,49,50]. Thus, the whole and probiotic pulp in Figure 3A,B presented, respectively, significant results at four different concentrations. Additionally, both pulps revealed antioxidant activity for concentrations beyond 0.05 μg/mL. Moreover, the antioxidant reaction is directly related to the assessed concentrations, thereby suggesting a dose-dependent reaction. The probiotic pulp presented an antioxidant action with similar differences between concentrations when compared with the whole pulp. This result suggests that the probiotic growth did not alter the antioxidant properties that can be evaluated through the TRAP assay.

Furthermore, by using the total antioxidant reactivity assay, it is possible to observe the free-radical luminescence effect when an antioxidant compound is added to the pulp. Thus, with this test, it is possible to determine the antioxidant quality available within the samples. Figure 3C,D present the TAR of both whole and probiotic caja pulps, respectively. The results reveal significance in both samples for concentrations above 0.5 μg/mL. Moreover, even though a higher level of TAR was detected at 50 μg/mL, no significant difference was found between the whole and probiotic pulps.

Therefore, our results suggest that the antioxidant activities are dependent on the chemical structure and the phytochemical concentration, such as phenolic compounds. These molecules remain stable during the fermentation, and thus the antioxidant features can be kept. Taken together, those results also indicate that the functional properties of the caja pulp can be improved via probiotic culture resulting in a functional product that can offer several health-specific benefits.

### 3.4. Probiotic Pulp Shelf-Life Study

Table 3 presents the characterization results performed during the storage period for both the probiotic pulp and the control medium. The fermented samples were stored for 28 days at 4 °C. The number of viable cells in the probiotic caja pulp and the control medium decreased during storage. It is noted when analyzing the data in Table 3 that at 14 days of storage, a more evident reduction in the number of bacteria is observed, this reduction being statistically significant. Similar products currently available on the market have a shelf life of around 15 days because, after that time, beverages fermented with live microorganisms show changes in quality. However, analyzing the number of viable bacteria in this study, it can be said that the quality of the product remains little changed in the 28 days of evaluation. Despite the reduction in the viability of the probiotic cells during the storage period, at the end of the 28 days, some viable cells greater than 10 Log CFU/mL were observed, which is considered adequate for fermented products containing probiotics [51].

The viability found in our work is superior to the values found for similar conditions in other studies, such as Silva et al. [52], while studying the viability of probiotic bacteria by complex coacervation. Similarly, Barat and Ozcan [53], when studying the growth of probiotic bacteria and characteristics of fermented milk containing fruit matrices during storage, samples of fermented milk containing red grape juice had a significantly (*p* < 0.05) greater number of *Streptococcus thermophilus* and *Lactobacillus acidophilus* cells, while *Lactobacillus delbrueckii* subsp*. Bulgaricus* and *Bifidobacterium animalis *subsp. *Lactis *cells were higher in fermented milk containing blackberry juice; the lowest levels of probiotics were found in fermented milk containing carnelian cherry. Shori [54], while reviewing the probiotic bacteria viability in dairy and non-dairy foods, cited a Favaro-Trindade et al. [55] work that studied the viability of an acerola probiotic ice cream. The authors found the stability of 14 days of storage with an approximate concentration of 6 Log CFU/mL. Barat e Ozcan [53] justifies the result by suggesting that the addition of fruit pulps for probiotic products guarantees more nutrients for bacteria multiplication.

The reduction in the number of cells was accompanied by a decrease in pH and an increase in the acidity of the probiotic caja pulp in the control sample until day 7 of storage. Some studies report that during storage, the pH of probiotic drinks decreases [12,28,51]. The decrease in pH during storage is related to the decrease in pH and the accumulation of acids in the medium [51]. This behavior observed in caja pulp during cold storage may be the result of the sum of the different post-acidification capacities of *Bifidobacterium animalis* ssp. *lactis* and the buffering power of the substrate [12]. In addition, dead cells can release enzymes that hydrolyze the sugars in fruit juice, thus lowering the pH, which also partly explains the reduction in total soluble solids [28], which decreased significantly on all evaluation days for the caja pulp.

Finally, the comparison between the caja pulp and the control medium showed that the caja pulp presents better results than the control medium, showing to be a suitable food matrix for the development of probiotic foods with *Bifidobacterium animalis* ssp*. lactis*, guaranteeing the delivery of probiotics in high concentrations, capable of benefiting the health of the consumer, and the product can be stored for up to 28 days in refrigerated conditions, guaranteeing the quality and food safety of the product.

## 4. Conclusions

In this study, the results suggest that caja pulp is an alternative and satisfactory medium for the development of a probiotic food obtained by fermentation with *Bifidobacterium animalis* ssp. *lactis*. The product obtained maintains the original health benefits, such as the antioxidant properties of the pulp, and, consequently, increases the benefits by using a probiotic bacterium. Based on the results presented, future studies on the metabolites resulting from fermentation and the study of conservation technologies, such as microencapsulation by spray dryer and/or spouted bed technique, can contribute to the development of high quality and safe probiotic foods based on cashew pulp.

## Figures and Tables

**Table 1:** Biological parameters of the growth of *Bifidobacterium animalis* ssp. *lactis* in caja pulp and the control medium (MRS broth).

Parameters	Probiotic caja Pulp	Control Medium
Número inicial de bactérias (LogCFU/mL)	10.00	10.00
Maximum number of bacteria (LogCFU/mL)	10.79	10.62
Maximum growth rate (1/h)	0.12	0.12
*lag* phase (h)	15	15
Generation time (h)	5.78	5.90

**Table 2:** Physicochemical characterization of the whole and probiotic caja pulp, fermented for 22 h.

Parameter	Whole Caja Pulp	Probiotic Caja Pulp **
pH	2.91 ± 0.03	6.03 ± 0.05 *
Acidity (g citric acid/100 mL)	1.72 ± 0.09	0.12 ± 0.02 *
Total soluble solids (°Brix)	11.83 ± 0.11	11.33 ± 0.28
Water content (%)	86.29 ± 0.13	87.53 ± 0.15
Total solids (%)	13.70 ± 0.13	12.46 ± 0.15
Ascorbic acid (mg/g)	15.84 ± 0.15	15.55 ± 0.05 *
Total phenolics (mg/g)	185.00 ± 6.14	185.26 ± 0.55
Carotenoids (mg/100 g)	0.023 ± 0.01	0.036 ± 0.01
Reducing carbohydrates (μg/mg)	3.63 ± 0.08	3.20 ± 0.01

The mean of three replicates (*n* = 3) and the values are expressed as the mean ± SEM. * *p* < 0.05 differs statistically when comparing the whole pulp with the probiotic pulp of caja in each parameter analyzed according to the Student *t*-test. ** The acidity of the probiotic pulp was calculated in g lactic acid/100 mL.

**Table 3:** Results of the physicochemical characterizations during the storage period of caja probiotic pulp and the control medium were obtained after 22 h of fermentation.

	Time (days)	pH	Acidity (g Lactic Acid/100 mL)	Total Soluble Solids (°Brix)	Number of Viable Cells (Log CFU/mL)
Probiotic caja pulp	1	6.03 ± 0.05 *	0.12 ± 0.01 *	11.83 ± 0.28 *	10.80 ± 0.05
	7	6.15 ± 0.05 *	0.18 ± 0.01 *	11.26 ±0.05 *	10.75 ± 0.02
	14	6.06 ± 0.05 *	0.17 ± 0.001 *	10.26 ± 0.11 *	10.37 ± 0.03 *
	21	5.76 ± 0.05 *	0.15 ± 0.01 *	10.23 ± 0.15 *	10.19 ± 0.01 *
	28	5.80 ± 0.10 *	0.13 ± 0.01 *	10.20 ±0.10 *	10.13 ± 0.02 *
Control medium	1	5.03 ± 0.05	0.53 ± 0.03	6.06 ± 0.11	10.71 ± 0.06
	7	4.76 ± 0.05 *	0.57 ± 0.01	5.96 ± 0.05	10.65 ± 0.08
	14	5.00 ± 0.01	0.54 ± 0.04	5.86 ± 0.11	10.41 ± 0.05 *
	21	5.10 ± 0.10	0.64 ± 0.01 *	5.86 ± 0.05	10.11 ± 0.03 *
	28	5.23 ± 0.05	0.43 ± 0.01 *	5.03 ± 0.05 *	10.04 ± 0.12 *

The mean of three replicates (*n* = 3) and the values are expressed as the mean ± SEM. * *p* < 0.05 differs statistically when comparing day 1 of the control medium with the other days of it and the probiotic caja pulp according to the Student *t*-test.

## Data Availability

The data presented in this study are available on request from the corresponding author.

## References

[1] Oladunjoye A.O., Adeboyejo F.O., Okekunbi T.A., Aderibigbe O.R. (2021). Effect of Thermosonication on Quality Attributes of Hog Plum (*Spondias mombin* L.) Juice. Ultrason. Sonochem..

[2] Aniceto A., Montenegro J., Cadena R.d.S., Teodoro A.J. (2021). Physicochemical Characterization, Antioxidant Capacity, and Sensory Properties of Murici (*Byrsonima crassifolia* (L.) Kunth) and Taperebá (*Spondias mombin* L.) Beverages. Molecules.

[3] de Assis R.C., Soares R.d.L.G., Siqueira A.C.P., de Rosso V.V., de Sousa P.H.M., Mendes A.E.P., Costa E.d.A., Carneiro A.P.d.G., Maia C.S.C. (2020). Determination of Water-Soluble Vitamins and Carotenoids in Brazilian Tropical Fruits by High Performance Liquid Chromatography. Heliyon.

[4] Oladunjoye A.O., Eziama S.C. (2020). Effect of Microwave-Assisted Alkaline Treatment on Physicochemical, Functional and Structural Properties of Hog Plum (*Spondias mombin* L.) Bagasse. LWT.

[5] Wang Y., Li H., Ren Y., Wang Y., Yaopeng R., Xiaowei W., Tianli Y., Zhouli W., Zhenpeng G. (2022). Preparation, Model Construction and Efficacy Lipid-Lowering Evaluation of Kiwifruit Juice Fermented by Probiotics. Food Biosci..

[6] Pereira B.L.B., Rodrigue A., Arruda F.C.d.O., Bachiega T.F., Lourenço M.A.M., Correa C.R., Azevedo P.S., Polegato B.F., Okoshi K., Fernandes A.A.H. (2020). *Spondias mombin* L. Attenuates Ventricular Remodelling after Myocardial Infarction Associated with Oxidative Stress and Inflammatory Modulation. J. Cell. Mol. Med..

[7] Brito S.A., Barbosa I.S., de Almeida C.L.F., de Medeiros J.W., Silva Neto J.C., Rolim L.A., da Silva T.G., Ximenes R.M., de Menezes I.R.A., Caldas G.F.R. (2018). Evaluation of Gastroprotective and Ulcer Healing Activities of Yellow Mombin Juice from *Spondias mombin* L. PLoS ONE.

[8] e Silva T.L.L., da Silva E.P., Asquieri E.R., Vieira E.C.S., Silva J.S., da Silva F.A., Damiani C. (2018). Physicochemical Characterization and Behavior of Biocompounds of Caja-Manga Fruit (*Spondias mombin* L.). Food Sci. Technol..

[9] Ojediran J.O., Okonkwo C.E., Olaniran A.F., Iranloye Y.M., Adewumi A.D., Erinle O., Afolabi Y.T., Adeyi O., Adeyi A. (2021). Hot Air Convective Drying of Hog Plum Fruit (*Spondias mombin*): Effects of Physical and Edible-Oil-Aided Chemical Pretreatments on Drying and Quality Characteristics. Heliyon.

[10] Rastogi Y.R., Thakur R., Thakur P., Mittal A., Chakrabarti S., Siwal S.S., Thakur V.K., Saini R.v., Saini A.K. (2022). Food Fermentation—Significance to Public Health and Sustainability Challenges of Modern Diet and Food Systems. Int. J. Food Microbiol..

[11] Paramithiotis S., Das G., Shin H.-S., Patra J.K. (2022). Fate of Bioactive Compounds during Lactic Acid Fermentation of Fruits and Vegetables. Foods.

[12] Fonseca H.C., Melo D.d.S., Ramos C.L., Menezes A.G.T., Dias D.R., Schwan R.F. (2021). Sensory and Flavor-Aroma Profiles of Passion Fruit Juice Fermented by Potentially Probiotic *Lactiplantibacillus plantarum* CCMA 0743 Strain. Food Res. Int..

[13] Paredes J.L., Escudero-Gilete M.L., Vicario I.M. (2022). A New Functional Kefir Fermented Beverage Obtained from Fruit and Vegetable Juice: Development and Characterization. LWT.

[14] Tripathi M.K., Giri S.K. (2014). Probiotic Functional Foods: Survival of Probiotics during Processing and Storage. J. Funct. Foods.

[15] Yerlikaya O., Saygili D., Akpinar A. (2021). An Application of Selected Enterococci Using *Bifidobacterium animalis* Subsp. *Lactis* BB-12 in Set-Style Probiotic Yoghurt-like Products. Food Biosci..

[16] Morais S.G.G., da Silva Campelo Borges G., dos Santos Lima M., Martín-Belloso O., Magnani M. (2019). Effects of Probiotics on the Content and Bioaccessibility of Phenolic Compounds in Red Pitaya Pulp. Food Res. Int..

[17] (2007). Informe Técnico No. 33.

[18] (2016). Official Methods of Analysis of AOAC International.

[19] (2006). Milk Products—Enumeration of Presumptive *Lactobacillus Acidophilus* on a Selective Medium—Colony-Count Technique at 37 °C.

[20] de Man J.C., Rogosa M., Sharpe M.E. (1960). A medium for the cultivation of lactobacilli. J. Appl. Bacteriol..

[21] Miller G.L. (1959). Use of Dinitrosalicylic Acid Reagent for Determination of Reducing Sugar. Anal. Chem..

[22] Keller T., Schwager H. (1977). Air Pollution and Ascorbic Acid. For. Pathol..

[23] Singleton V.L., Orthofer R., Lamuela-Raventós R.M. (1999). [14] Analysis of Total Phenols and Other Oxidation Substrates and Antioxidants by Means of Folin-Ciocalteu Reagent. Methods Enzymol..

[24] Lichtenthaler H.K. (1987). [34] Chlorophylls and Carotenoids: Pigments of Photosynthetic Biomembranes. Methods Enzymol..

[25] GraphPad Prisma Version 8.0.0 for Windows, San Diego, CA, USA. www.graphpad.com.

[26] Moresco K., Silveira A., Schnorr C., Zeidán-Chuliá F., Bortolin R., Bittencourt L., Mingori M., Heimfarth L., Rabelo T., Morrone M. (2017). Supplementation with Achyrocline Satureioides Inflorescence Extracts to Pregnant and Breastfeeding Rats Induces Tissue-Specific Changes in Enzymatic Activity and Lower Neonatal Survival. Biomedicines.

[27] Olivares A., Soto C., Caballero E., Altamirano C. (2019). Survival of Microencapsulated *Lactobacillus casei* (Prepared by Vibration Technology) in Fruit Juice during Cold Storage. Electron. J. Biotechnol..

[28] Andrade R., Santos E., Azoubel P., Ribeiro E. (2019). Increased Survival of Lactobacillus Rhamnosus ATCC 7469 in Guava Juices with Simulated Gastrointestinal Conditions during Refrigerated Storage. Food Biosci..

[29] (1972). Minitab Satatistical Software.

[30] Maier R.M., Pepper I.L. (2015). Bacterial Growth. Environmental Microbiology.

[31] Baranyi J., Roberts T.A. (1994). A Dynamic Approach to Predicting Bacterial Growth in Food. Int. J. Food Microbiol..

[32] Mustafa S.M., Chua L.S., El-Enshasy H.A., Abd Majid F.A., Hanapi S.Z. (2020). Kinetic Profile and Anti-Diabetic Potential of Fermented Punica Granatum Juice Using *Lactobacillus casei*. Process Biochem..

[33] Jaiswal A.K., Abu-Ghannam N. (2013). Kinetic Studies for the Preparation of Probiotic Cabbage Juice: Impact on Phytochemicals and Bioactivity. Ind. Crops Prod..

[34] Solval K.M., Chouljenko A., Chotiko A., Sathivel S. (2019). Growth Kinetics and Lactic Acid Production of *Lactobacillus plantarum* NRRL B-4496, *L. acidophilus* NRRL B-4495, and *L. reuteri* B-14171 in Media Containing Egg White Hydrolysates. LWT.

[35] Markkinen N., Laaksonen O., Nahku R., Kuldjärv R., Yang B. (2019). Impact of Lactic Acid Fermentation on Acids, Sugars, and Phenolic Compounds in Black Chokeberry and Sea Buckthorn Juices. Food Chem..

[36] Panghal A., Janghu S., Virkar K., Gat Y., Kumar V., Chhikara N. (2018). Potential Non-Dairy Probiotic Products—A Healthy Approach. Food Biosci..

[37] Hernández N.B.S. (2004). Evaluación de Leche de Cabra Como Sustrato Para El Desarrollo de Un Probiótico Fermentado Con Bifidobacterium Infantis y Bacterias Ácido Lácticas e Implementación de Un Método Para Identificar, B. Infantis Mediante Reacción En Cadena de La Polimerasa (PCR).

[38] Zeybek N., Rastall R.A., Buyukkileci A.O. (2020). Utilization of Xylan-Type Polysaccharides in Co-Culture Fermentations of *Bifidobacterium* and *Bacteroides* Species. Carbohydr. Polym..

[39] Usta-Gorgun B., Yilmaz-Ersan L. (2020). Short-Chain Fatty Acids Production by *Bifidobacterium* Species in the Presence of Salep. Electron. J. Biotechnol..

[40] Asad J., Jacobson A.F., Estabrook A., Smith S.R., Boolbol S.K., Feldman S.M., Osborne M.P., Boachie-Adjei K., Twardzik W., Tartter P.I. (2008). Does Oncotype DX Recurrence Score Affect the Management of Patients with Early-Stage Breast Cancer?. Am. J. Surg..

[41] Wang Z., Feng Y., Yang N., Jiang T., Xu H., Lei H. (2022). Fermentation of Kiwifruit Juice from Two Cultivars by Probiotic Bacteria: Bioactive Phenolics, Antioxidant Activities and Flavor Volatiles. Food Chem..

[42] Filannino P., Cavoski I., Thligene N., Vincentini O., de Angelis M., Silano M., Gobbetti M., di Cagno R. (2016). Correction: Lactic Acid Fermentation of Cactus Cladodes (*Opuntia ficus-indica* L.) Generates Flavonoid Derivatives with Antioxidant and Anti-Inflammatory Properties. PLoS ONE.

[43] Hashemi S.M.B., Jafarpour D. (2020). Fermentation of Bergamot Juice with *Lactobacillus Plantarum* Strains in Pure and Mixed Fermentations: Chemical Composition, Antioxidant Activity and Sensorial Properties. LWT.

[44] Wu Y., Li S., Tao Y., Li D., Han Y., Show P.L., Wen G., Zhou J. (2021). Fermentation of Blueberry and Blackberry Juices Using *Lactobacillus plantarum*, *Streptococcus thermophilus* and *Bifidobacterium bifidum*: Growth of Probiotics, Metabolism of Phenolics, Antioxidant Capacity in Vitro and Sensory Evaluation. Food Chem..

[45] de la Fuente B., Luz C., Puchol C., Meca G., Barba F.J. (2021). Evaluation of Fermentation Assisted by Lactobacillus Brevis POM, and Lactobacillus Plantarum (TR-7, TR-71, TR-14) on Antioxidant Compounds and Organic Acids of an Orange Juice-Milk Based Beverage. Food Chem..

[46] Maldonado-Astudillo Y.I., Alia-Tejacal I., Núñez-Colín C.A., Jiménez-Hernández J., Pelayo-Zaldívar C., López-Martínez V., Andrade-Rodríguez M., Bautista-Baños S., Valle-Guadarrama S. (2014). Postharvest Physiology and Technology of *Spondias purpurea* L. and *S. mombin* L. Sci. Hortic..

[47] Peng W., Meng D., Yue T., Wang Z., Gao Z. (2021). Effect of the Apple Cultivar on Cloudy Apple Juice Fermented by a Mixture of *Lactobacillus acidophilus*, *Lactobacillus plantarum*, and *Lactobacillus fermentum*. Food Chem..

[48] Bernini L.J., Simão A.N.C., de Souza C.H.B., Alfieri D.F., Segura L.G., Costa G.N., Dichi I. (2018). Effect of *Bifidobacterium lactis* HN019 on Inflammatory Markers and Oxidative Stress in Subjects with and without the Metabolic Syndrome. Br. J. Nutr..

[49] Martins A.N.A., Pasquali M.A.d.B., Schnorr C.E., Martins J.J.A., de Araújo G.T., Rocha A.P.T. (2019). Development and Characterization of Blends Formulated with Banana Peel and Banana Pulp for the Production of Blends Powders Rich in Antioxidant Properties. J. Food Sci. Technol..

[50] Mattietto R.A., Matta V.M. (2011). Cajá (*Spondias mombin* L.). Postharvest Biology and Technology of Tropical and Subtropical Fruits.

[51] Pereira A.L.F., Maciel T.C., Rodrigues S. (2011). Probiotic Beverage from Cashew Apple Juice Fermented with *Lactobacillus Casei*. Food Res. Int..

[52] da Silva T.M., de Deus C., de Souza Fonseca B., Lopes E.J., Cichoski A.J., Esmerino E.A., de Bona da Silva C., Muller E.I., Moraes Flores E.M., de Menezes C.R. (2019). The Effect of Enzymatic Crosslinking on the Viability of Probiotic Bacteria (*Lactobacillus acidophilus*) Encapsulated by Complex Coacervation. Food Res. Int..

[53] Barat A., Ozcan T. (2018). Growth of Probiotic Bacteria and Characteristics of Fermented Milk Containing Fruit Matrices. Int. J. Dairy Technol..

[54] Shori A.B. (2015). The Potential Applications of Probiotics on Dairy and Non-Dairy Foods Focusing on Viability during Storage. Biocatal. Agric. Biotechnol..

[55] Favaro-Trindade C.S., Bernardi S., Bodini R.B., Balieiro J.C.D.C., de Almeida E. (2006). Sensory Acceptability and Stability of Probiotic Microorganisms and Vitamin C in Fermented Acerola (*Malpighia emarginata* DC.) Ice Cream. J. Food Sci..

